# Exploration of Cannabis Use with Excessive Social Media Use Among U.S. College Students

**DOI:** 10.3390/bs15111455

**Published:** 2025-10-25

**Authors:** Madelyn J. Hill, Rebecca A. Vidourek, Keith A. King, Matthew Lee Smith, Ashley L. Merianos

**Affiliations:** 1 Department of Social and Public Health, College of Health Sciences and Professions, Ohio University, Grover Center W379, Athens, OH 45701, USA; hill4mj@mail.uc.edu; 2 School of Human Services, University of Cincinnati, Cincinnati, OH 45221, USA; rebecca.vidourek@uc.edu (R.A.V.); keith.king@uc.edu (K.A.K.); 3 Department of Health Behavior, School of Public Health, Texas A&M University, College Station, TX 77845, USA; matthew.smith@tamu.edu

**Keywords:** cannabis use, cannabis use risk, excessive social media use, college students

## Abstract

Excessive social media use (ESMU) may negatively impact college students. Less is known about whether cannabis use may influence ESMU. This study assessed the association between current cannabis use and ESMU in addition to cannabis use risk and ESMU among U.S. college students. An analysis of the 2022–2023 American College Health Association’s National College Health Assessment dataset including 65,052 college students aged 18–24-years old was performed. Unadjusted and adjusted logistic regression models were analyzed. In total, 23.2% of college students reported current cannabis use within the past 30 days. The unadjusted and adjusted logistic model results indicated that college students reporting current cannabis use were at increased odds of reporting ESMU compared to college students not reporting current cannabis use (OR = 1.22, 95% CI = 1.16–1.28; AOR = 1.27, 95% CI = 1.20–1.34, respectively). Furthermore, unadjusted and adjusted logistic regression results demonstrated that college students with moderate cannabis use risk (OR = 1.21, 95% CI = 1.12–1.30; AOR = 1.21, 95% CI = 1.12–1.30) or high cannabis use risk (OR = 1.78, 95% CI = 1.50–2.11; AOR = 1.82, 95% CI = 1.53–2.16) were at increased odds of reporting ESMU compared to college students with low cannabis use risk. U.S. college students who currently use cannabis, especially those exhibiting a moderate or high cannabis use risk, are at an increased odds of excessively using social media sites.

## 1. Introduction

Online platforms, known as social media, provide an outlet where users can share personal content, opinions, and ideas with other users. In 2024, young adults ages 18–24 years averaged six hours a day online with the majority of time spent on social media platforms (Field, 2024). Instagram, Snapchat, YouTube, and TikTok are among the most used social media platforms reported by young adults (Field, 2024; Gottfried, 2024). Motivations for social media use include information seeking, personal expression, connecting with established social networks, for entertainment purposes, or to alleviate boredom (Khan, 2017; Quan-Haase & Young, 2010; Stockdale & Coyne, 2020). Social media platforms can also motivate and encourage use through design choices that exploit users’ self-control and emphasize prolonged use (Ichihashi & Kim, 2023). For example, one of these design choices, infinite scrolling, allows users to continually scroll without needing to click “next page” which works to keep users on social media platforms (Cara, 2019).

Although not inherently negative, when used in excess, social media can become harmful to the user (Przybylski, 2014; Przybylski & Weinstein, 2017). Previous research has indicated that users tend to report poorer outcomes with social media use of ≥2 h/day, or excessive social media use (ESMU). ESMU is defined as being strongly concerned with and motivated to use social media to the point of impeding other responsibilities and psychological well-being (Andreassen & Pallesen, 2014; Primack et al., 2017; Sampasa-Kanyinga & Lewis, 2015). Consequently, approximately 25% of young adults ages 19–30 years old reported ESMU (Primack et al., 2017). Social media use, especially when used in excess, is associated with social isolation (Primack et al., 2017), loneliness (Song et al., 2014; Wang et al., 2024), poorer sleep outcomes (Baiden et al., 2019; Levenson et al., 2016), physical inactivity (Shimoga et al., 2019), and decreased mental health (Andreassen et al., 2017; Lin et al., 2016; Vannucci et al., 2017). In addition to the physical and mental effects, social media and internet use are associated with an increase in experimentation and use of substances, such as cannabis (Doggett et al., 2019; Nagata et al., 2025).

Cannabis, also known as marijuana, refers to the dried leaves, stems, and seeds of the Cannabis plant (National Academies of Sciences, Engineering, and Medicine, 2024). The cannabis plant is a psychoactive drug that is comprised of over 100 cannabinoids, with the main being delta-9-tetrahydrocannabinol (THC), cannabidiol, and cannabinol (Kinghorn et al., 2017; National Academies of Sciences, Engineering, and Medicine, 2024). The most common methods of cannabis use among young adults include smoking, electronic vaping, or infused food or drinks (Wadsworth et al., 2022). Through inhalation or ingestion, the cannabinoid THC elicits feelings of relaxation, euphoria, and intoxication and can encourage repetitive use (Martin-Santos et al., 2012). Consequently, cannabis use among college students has significantly increased within the past decade (Patrick et al., 2024a). In 2023, 26.1% of college students reported using cannabis within the past 30 days, with 6.3% of college students reporting daily cannabis use (Patrick et al., 2024a). Among college students, frequency of cannabis use is predicted by motivations to help with sleep, for coping, or for enjoyment (Halter & Abar, 2023).

Continued use of cannabis can lead to dependence or cannabis use disorder (American Psychiatric Association, 2013; Connor et al., 2021), which impacts approximately 30% of all cannabis users in the U.S. (Hasin et al., 2015). Factors such as duration of cannabis use, increases in amount used, inability to stop use despite physical or social risks, and physiological dependence can all indicate cannabis use disorder (American Psychiatric Association, 2013; Connor et al., 2021). The risks for cannabis use disorder and increases in cannabis use among college students are concerning given the attributed health effects. Research has suggested that cannabis use is linked to chronic respiratory conditions (Kempker et al., 2015; Tashkin et al., 2012), cardiovascular diseases (Kalla et al., 2018; Ladha et al., 2021), psychosis (Di Forti et al., 2015; Wright et al., 2021), and suicidal thoughts or behaviors (Ahuja et al., 2022; Vidal et al., 2023). Additionally, cannabis use is associated with experiencing feelings of amotivation described as apathy, lower ability to concentrate, and reduced motivation for goal-directed behaviors (Mcglothlin & West, 1968; Volkow et al., 2016). The feelings of amotivation generated from cannabis can impede academic achievements of college students through lower GPAs (Suerken et al., 2016), reduced class attendance (Arria et al., 2015), and delayed time to graduation (Suerken et al., 2016). Beyond the physical and mental effects of cannabis, the frequency of use can increase the odds of engaging in sedentary screen time behaviors, such as watching television, using the internet, or playing video games among individuals ≥ 12 years old (Neilson & Lin, 2019).

Based on the high prevalence of both cannabis use and sedentary screen time behaviors among college students (Field, 2024; Patrick et al., 2024a; Primack et al., 2017), a potential relationship between cannabis use and ESMU should be explored. Research has previously explored cannabis with social media behaviors among young adults, such as posting and engaging with cannabis related content on social media (Cavazos-Rehg et al., 2016, 2018; Moreno et al., 2018). Additionally, the relationship between cannabis advertising on social media and cannabis use has previously been explored indicating that exposure to cannabis advertising is related to cannabis use among young adults (Cabrera-Nguyen et al., 2016; Krauss et al., 2017; Noel et al., 2024). Similarly, another study suggested that social media predicts cannabis use. A study by Gutierrez and Cooper (2016) explored time spent on social media sites predicting use of cannabis, alcohol, and synthetic cannabinoids among college students. Findings indicated that time spent on social media was not associated with cannabis use, yet alcohol and synthetic cannabinoid use were associated (Gutierrez & Cooper, 2016). The lack of relationship between time spent on social media and cannabis use could be attributed to participants underreporting substance use (Gutierrez & Cooper, 2016). Irrespective of the direction, there is minimal literature about the potential association between cannabis use and ESMU among college students. To explore this association, this study applied the theory of compensatory internet use, which focuses on the frequency of online social media use as an escape mechanism (Kardefelt-Winther, 2014a). Cannabis use has similarly been linked to feelings of wanting to escape life problems among older adolescents (Patrick et al., 2024b). Cannabis use could potentially be a precursor to excessive social media use as a means to further escape (Kardefelt-Winther, 2014a, 2014b; Patrick et al., 2024b). Thus, this study assessed the directionality of cannabis use predicting ESMU.

Within the U.S., cannabis legislation has greatly changed over the past decade, with 24 U.S. states with legalized recreational cannabis in 2024 (Schaeffer, 2024). Given these changes in legislation, social norms surrounding cannabis use may have progressed, potentially contributing to participant’s comfortability disclosing use (Clarke et al., 2018; Wen et al., 2019; Winfield-Ward & Hammond, 2024). Additionally, social media platforms have changed since the aforementioned study was published in 2016, potentially leading to an increase in social media use. The video-based social media platform, TikTok, reported one-third of U.S. adults as users in 2023, which is a 12% increase from 2021 (Gottfried, 2024). Furthermore, cannabis use risk, such as duration of use and inability to stop use, has not been explored with ESMU. The concurrent use of cannabis and ESMU could place college students at increased risk for negative health outcomes warranting further investigation into this potential relationship.

To address the gap in the literature regarding the relationship between cannabis use and ESMU, the purpose of this study was to explore the association between current cannabis use and ESMU among U.S. college students. The relationship between cannabis use risk and ESMU was also assessed among U.S. college students. It was hypothesized that U.S. college students who reported current cannabis use within the past 30 days would be at increased odds of reporting ESMU compared to U.S. college students who did not report current cannabis use. It was also hypothesized that U.S. college students with a moderate or high cannabis use risk would be at increased odds of reporting ESMU compared to U.S. college students with a low cannabis use risk.

## 2. Materials and Methods

A secondary data analysis was conducted using the 2022–2023 American College Health Association’s (ACHA’s) National College Health Assessment (NCHA) dataset. The NCHA is a cross-sectional survey that collects health and wellness data of college students in the U.S. The ACHA-NCHA-III survey was used in this study. Developed in 2019, the ACHA-NCHA-III provides a comprehensive tool to measure college students’ health behaviors and outcomes (Lederer & Hoban, 2022). There were 53 postsecondary institutions that self-selected to participate in the biannual ACHA-NCHA-III fall 2022 online survey (American College Health Association, 2022) and 127 postsecondary institutions that self-selected to participate in the spring 2023 online survey (American College Health Association, 2023). Cumulatively, there were 114,829 completed ACHA-NCHA-III surveys between the Fall 2022 (N = 35,692) and Spring 2023 (N = 79,137) surveys (American College Health Association, 2022, 2023). In the Fall 2022 survey, 31.7% of institutions were based in southern U.S. states, and the Spring 2023 survey had 44.8% of institutions based in western U.S. states (American College Health Association, 2022, 2023). Responses from undergraduate college students, ages 18–24 years old, who attend a four-year institution were included in this study (*n* = 65,052). This research was determined to be non-human subjects research by a university based Institutional Review Board due to the lack of identifiable information included in the ACHA-NCHA public use datasets.

For this study, two measures were used to assess cannabis use among college students. The ACHA-NCHA-III measured cannabis use by asking participants, “When, if ever, was the last time you used cannabis/marijuana?” This measure included medical and non-medical use of cannabis. Response options included “within the last 2 weeks,” “more than 2 weeks ago but within the last 30 days,” “more than 30 days ago but within the last 3 months,” “more than 3 months ago but within the last 12 months,” “more than 12 months ago,” and “never.” For this study, current cannabis use was defined as reporting cannabis use within the past 30 days.

Additionally, the ACHA-NCHA-III included the Alcohol, Smoking and Substance Involvement Screening Test (ASSIST) (World Health Organization, 2010), which was asked among a sub-sample of participants in this study (*n* = 25,431). Including eight-items, the ASSIST covers frequency of use and risks associated with cannabis use (World Health Organization, 2010). Participants were asked, “In your life which of the following substances have you ever used (non-medical use only)?” Those who selected cannabis were included in the analyses. The frequency of cannabis use was measured by asking participants, “In the past three months how often have you used the substances you mentioned?” with response options of “never,” “once or twice,” “monthly,” “weekly,” and “daily/almost daily.” The risks of cannabis use were measured through items, “During the past three months how often have you had a strong desire or urge to use (drug)?,” “During the past three months, how often has your use of (drug) led to health, social, legal or financial problems?,” and “During the past three months how often have you failed to do what was normally expected of you because of your use of (drug)?” with response options of “never,” “once or twice,” “monthly,” “weekly,” and “daily/almost daily.” Additional cannabis risk items on the ASSIST included, “Has a friend or relative or anyone else ever express concern about your use of (drug)?,” “Have you ever tried to cut down or stop using (drug) but failed?,” and “Have you ever used any drug by injection?” with response options including “no, never,” “yes, in the past three months,” and “yes, but not in the past three months.” Based on participants’ ASSIST responses, a Substance Specific Involvement Score (SSIS) was calculated and ranged from 0–39, with higher scores indicating a higher level of risk associated cannabis use. A sub-sample of 25,431 participants had these scores available for analysis. The SSIS cannabis score was calculated based on responses to the previously described screening, frequency, and risk questions on the ASSIST by the ACHA-NCHA-III. Responses were summed and collapsed into three categories for analysis: (1) low cannabis use risk (scores 0–3); (2) moderate cannabis use risk (scores 4–26); and (3) high cannabis use risk (scores 27–39) (American College Health Association, 2022, 2023; McNeely et al., 2016; World Health Organization, 2010). The SSIS cannabis score indicates the participant’s need for intervention for cannabis use. Low cannabis use risk is viewed as minimal need for intervention, moderate cannabis use risk indicates the need for a brief intervention, and high cannabis use risk suggests a high risk for dependence highlighting the participant’s need for a referral to a specialist, assessment, or treatment for cannabis use (World Health Organization, 2010).

Frequency of social media use was measured through one-item on the ACHA-NCHA-III survey. Participants were asked, “How many hours do you spend in a typical week (7 days) on social media?” Responses included “0 h,” “1–5 h,” “6–10 h,” “11–15 h,” “16–20 h,” “21–25 h,” “26–30 h,” and “30 h.” The social media use responses were collapsed into binary choices for analyses: (1) no ESMU for participants that reported using social media for <16 h/week; and (2) ESMU for participants that reported using social media for ≥16 h/week. Due to pre-determined social media frequency categories created by ACHA-NCHA III, 16–20 h was used as the cut off for the binary categories. Including “16–20 h,” “21–25 h,” “26–30 h,” and “30 h” for the ESMU category meets or exceeds the 14 h of weekly social media use (i.e., ≥2 h daily) that is defined as excessive use (Andreassen & Pallesen, 2014; Primack et al., 2017; Sampasa-Kanyinga & Lewis, 2015).

This study included the following sociodemographic characteristics as covariates: age, sex (male and female), race and ethnicity (non-Hispanic White, non-Hispanic Black, non-Hispanic Asian, non-Hispanic Other/Multiracial, and Hispanic), and parent’s highest education level (≤high school graduate or equivalent, some college, undergraduate degree, and graduate degree). Other potential covariates included college-related factors: undergraduate year (first year, second year, third year, ≥fourth year), enrollment status (full-time and part-time), class format (entirely in-person, hybrid, and entirely online), current residence (campus housing, off-campus housing, and family member’s home), and member of a fraternity or sorority (yes/no).

Descriptive statistics were calculated for participant characteristics using SPSS (version 30.0). Chi-square analyses were conducted to determine the differences across ESMU categories for the categorical covariates (e.g., sex, race and ethnicity) and current cannabis use. An unadjusted logistic regression was modeled to examine the association between current cannabis use and ESMU with no current cannabis use being the reference group. Unadjusted odds ratios (ORs), 95% confidence intervals (CIs), and *p*-values were calculated. An adjusted logistic regression was modeled to examine this relationship while adjusting for the covariates (i.e., age, sex, race and ethnicity, parent’s highest education level, undergraduate year, enrollment status, class format, current residence, and fraternity or sorority membership). The adjusted ORs (AORs), 95% CIs, and *p*-values were calculated.

Among a sub-sample of 25,431 participants who had calculated SSIS cannabis scores, we conducted an unadjusted logistic regression model to assess the association between SSIS cannabis scores and ESMU with low cannabis use risk as the reference group. The ORs, 95% CIs, and *p*-values were calculated and presented. An adjusted logistic regression model was then conducted to assess the relationship between SSIS cannabis scores and ESMU, while adjusting for the covariates (i.e., age, sex, race and ethnicity, parent’s highest education level, undergraduate year, enrollment status, class format, current residence, and fraternity or sorority membership). The AORs, 95% CIs, and *p*-values were calculated and presented. All analyses used *p* < 0.05 to indicate statistical significance.

## 3. Results

Of the 65,052 participants included in this study, 71.5% were female and 56.9% were non-Hispanic White (Table 1). On average, participants were 19.9 (±1.4) years old with 31.7% being first year students, 24.8% being second year students, 24.3% being third year students, and 19.2% being ≥ fourth year students. Most respondents (96.3%) were enrolled in full-time classes and 66.0% were enrolled in entirely in-person classes. Approximately 7.7% of participants were a member of a fraternity or sorority. Over half of the participants (52.0%) reported living in campus housing. Additionally, over one-third of participants (36.4%) had a parent with a graduate degree education (see Table 1). A total of 23.2% (*n* = 15,084) of college students reported current cannabis use. Of the sub-sample with cannabis use risk scores (*n* = 25,431), 46.9% were low risk (*n* = 11,930), 49.7% were at moderate risk (*n* = 12,628), and 3.4% were at high risk (*n* = 873).

### 3.1. Participant Characterisics Based on Current Cannabis Use

Chi-square results found that all covariates, with the exception of enrollment status, significantly differed based on current cannabis use (see Table 1). Specifically, female participants reported a higher percentage of current cannabis use compared to male participants. Those who were non-Hispanic Other/Multiracial and non-Hispanic White had higher percentages of current cannabis use compared to the other racial and ethnic groups. For undergraduate year, as school year increased, current cannabis use increased. Participants enrolled in entirely in-person classes and in a fraternity or sorority had higher percentages of current cannabis use compared to participants enrolled in entirely in online or hybrid classes, and were not in a fraternity or a sorority. Participants who lived in off-campus housing had a higher percentage of current cannabis use compared to participants who lived in campus housing or a family member’s home. For parent’s education level, as education level increased, current cannabis use increased (see Table 1).

### 3.2. Current Cannabis Use and ESMU

ESMU (i.e., using social media for ≥16 h/week) was reported by 13.2% of participants. The unadjusted logistic regression model results demonstrated that college students currently using cannabis were at a 1.22 times higher odds of ESMU (95% CI = 1.16–1.28) compared to college students who are not currently using cannabis (Table 2).

While adjusting for the covariates, the adjusted logistic regression model results indicated that current cannabis users were at 1.27 times higher odds of ESMU (95% CI = 1.20–1.34) compared to non-current cannabis users (see Table 2). Within the adjusted logistic regression model results, participant age, sex, race and ethnicity, enrollment status, class format, fraternity or sorority membership, current residence, and parent’s education level were significantly associated with ESMU among current cannabis users. Female participants (AOR = 1.06, 95% CI = 1.00–1.11) and those who were non-Hispanic Black (AOR = 1.49, 95% CI = 1.35–1.65), non-Hispanic Asian (AOR = 1.15, 95% CI = 1.07–1.23), and non-Hispanic Other/Multiracial (AOR = 1.09, 95% CI = 1.02–1.17) were at increased odds of reporting ESMU compared to participants who were male and non-Hispanic White, respectively. Participants enrolled in part-time classes (AOR = 1.17, 95% CI = 1.05–1.32) and who had entirely online classes (AOR = 1.60, 95% CI = 1.40–1.83) or hybrid classes (AOR = 1.35, 95% CI = 1.29–1.42) were at significantly increased odds of reporting ESMU compared to participants enrolled in full-time classes and who had entirely in-person classes, respectively. Participants who were a member of a fraternity or a sorority (AOR = 1.20, 95% CI = 1.10–1.30) and who lived in off-campus housing (AOR = 1.06, 95% CI = 1.00–1.13) or a family member’s home (AOR = 1.18, 95% CI = 1.11–1.27) were at significantly increased odds of reporting ESMU compared to participants who were not a member of a fraternity or a sorority and who lived in campus housing, respectively. Participants with parents who had an education of some college (AOR = 1.23, 95% CI = 1.15–1.32), an undergraduate degree (AOR = 1.23, 95% CI = 1.15–1.32), and graduate degree (AOR = 1.18, 95% CI = 1.11–1.25) were at significantly increased odds of reporting ESMU than participants with parents who had an education of ≤high school graduate or equivalent. Conversely, participants who were older were at reduced odds of reporting ESMU (AOR = 0.97, 95% CI = 0.94–0.99) (see Table 2).

### 3.3. Current Use Risk and ESMU

When assessing cannabis use risk and ESMU, the unadjusted logistic regression model indicated that compared to participants with low cannabis use risk, participants with moderate cannabis use risk (OR = 1.21, 95% CI = 1.12–1.30) and high cannabis use risk (OR = 1.78, 95% CI = 1.50–2.11) were at increased odds of ESMU (Table 3). Similarly, the adjusted logistic regression model results indicated that college students with moderate cannabis use risk (AOR = 1.21, 95% CI = 1.12–1.30) and high cannabis use risk (AOR = 1.82, 95% CI = 1.53–2.16) were at an increased odds of ESMU compared to college students with low cannabis use risk, while adjusting for the covariates. The covariates of participant age, race and ethnicity, class format, fraternity or sorority membership, current residence, and parent’s education level were associated with ESMU (see Table 3). Participants who were non-Hispanic Black (AOR = 1.52, 95% CI = 1.28–1.80) or non-Hispanic Asian (AOR = 1.17, 95% CI = 1.03–1.32) were more likely to report ESMU compared to non-Hispanic White participants. Participants who had classes entirely online (AOR = 1.74, 95% CI = 1.40–2.15) and hybrid (AOR = 1.38, 95% CI = 1.28–1.49) were more likely to report ESMU compared to participants who had classes entirely in-person. Participants who were a fraternity or sorority member (AOR = 1.18, 95% CI = 1.06–1.33) or lived in a family member’s home (AOR = 1.24, 95% CI = 1.10–1.39) were more likely to report ESMU compared to participants who were not a member of fraternity or sorority or lived in campus housing, respectively. Participants with parents who had some college (AOR = 1.20, 95% CI = 1.07–1.33), an undergraduate degree (AOR = 1.17, 95% CI = 1.05–1.30), and a graduate degree (AOR = 1.13, 95% CI = 1.04–1.24) were more likely to report ESMU compared to participants with parents who had an education of ≤high school graduate or equivalent (see Table 3).

## 4. Discussion

This is among the first studies to investigate the relationship between cannabis use and ESMU among U.S. college students. More specifically, current cannabis use and cannabis use risk were assessed with ESMU defined as using social media ≥ 2 h/day. About 13% of participants reported ESMU, which is less than the previously reported 25.5% in young adults ages 19–30 years old (Primack et al., 2017). In this study, about 23% of college students currently used cannabis within the past 30 days, which is comparable to the previously reported prevalence among college students of 26% (Patrick et al., 2024a). Of those with cannabis use risk scores, about 47% were low risk, 50% were at moderate risk, and 3% were at high risk. In support of the study’s hypothesis, results demonstrate that reporting current cannabis use increased the odds of reporting ESMU among U.S. college students. Due to the novelty of this association, there is limited literature to support these findings among this at-risk population. This aligns with past research that showed time spent on social media and the internet were associated with cannabis use among youth and early adolescents (Doggett et al., 2019; Nagata et al., 2025). Conversely, previous findings have reported current cannabis use was not associated with time spent on social media platforms (Gutierrez & Cooper, 2016). Given the aforementioned study was conducted nearly a decade ago and synthetic cannabinoid use was found to be associated with increased time spent on social media, the current study’s results could indicate potential changes in college students’ comfortability disclosing cannabis use as well as increased accessibility. In 2016, there were eight U.S. states with legalized recreational cannabis, whereas in 2022 there were 19 U.S. states (Hayes, 2022; Steinmetz, 2016). Legalization of recreational cannabis can increase the rates of cannabis use among college students and potentially change their societal norms (Bae & Kerr, 2020; Barker & Moreno, 2021; Mennis et al., 2023; Miller et al., 2017). Furthermore, the role of social media to be entertainment and alleviate boredom, combined with the increased availability and feelings of escapism among cannabis users could potentially explain the relationship with ESMU displayed in this study. Additional mixed-methods original research can expand on these preliminary findings and determine reasons for concurrent use of cannabis and ESMU. These findings emphasize the need for interventions and messaging to generate awareness about cannabis and social media use among U.S. college students.

The hypothesis of this study related to the SSIS cannabis scores was also supported as U.S. college students with a moderate or high cannabis use risk were at an increased odds of reporting ESMU compared to college students with a low cannabis use risk. Within the logistic regression model, respondents with a high cannabis use risk had an 82% increased odds of reporting ESMU compared to low cannabis use risk, while controlling for the covariates. These results demonstrate that as cannabis use risk increases, the odds of ESMU increases among college students. This study provides preliminary evidence for a relationship between the severity of cannabis use and ESMU; however, more research is necessary to extensively investigate this relationship. As previously speculated, the amotivation of cannabis and feelings of escapism could lead users to use social media as a means to fulfill boredom, and based on these results cannabis use risk can amplify the odds of ESMU. With high cannabis risk users having increased odds of using social media excessively, social media platforms are a convenient way to reach this population for both cannabis use disorder and ESMU preventive interventions (Willoughby et al., 2024).

In the model results of current cannabis use, the sociodemographic covariates including participant age, sex, race and ethnicity, and parent’s highest education level were found to be predictors of ESMU among U.S. college students. Similarly, in the model results of cannabis use risk, the covariates of participant age, race and ethnicity, and parent’s education level were associated with ESMU. In the current cannabis use model, female college students were at an increased odds of reporting ESMU compared to male students. Based on previous research, female adolescents and young adults typically use social media platforms more frequently and at higher percentages than male adolescents and young adults (Castrén et al., 2022; Scott et al., 2017). In the current cannabis use model, non-Hispanic Black, non-Hispanic Asian, and non-Hispanic Other/Multicultural college students were at increased odds of reporting ESMU compared to non-Hispanic White students. In the cannabis use risk model, non-Hispanic Black and non-Hispanic Asian participants had increased odds of reporting ESMU compared to non-Hispanic White participants. Previous research has indicated that Black teenagers use social media and the internet at higher percentages than their White counterparts (Faverio & Sidoti, 2024; Rideout et al., 2022). However, there is limited research investigating social media use among Asian Americans, despite Asian Americans accessing the internet at higher percentages than other racial groups (Spooner, 2001). More research, similar to Charmaraman et al. (2018) that differentiates between Asian groups and multi-cultural participants is essential to further understand this relationship among college students.

In both the current cannabis use and cannabis use risk models, college students with a parent who had some college education, an undergraduate degree, or a graduate degree were at increased odds of reporting ESMU compared to users with a parent education level of a ≤high school graduate or equivalent. Parent education level can be used as a proxy for socioeconomic status, and was the only indicator available in the NCHA dataset. These study’s results are contrary to those indicated in international adolescents, where a lower socioeconomic status was related to excessive internet use (Faltýnková et al., 2020). Additionally, a study among Hispanic college students reported that father’s education level was not associated with ESMU; however, mother’s education level was not assessed (Mathis et al., 2022). Results from the current study could potentially indicate that college students with a higher parent education level may have more smartphones, tablets, or computers to access social media platforms (Faverio & Sidoti, 2024). The potential increased access to technology for students with a higher parent education level may have helped create a routinized technology behavior, and thus a positive perception of technology. It could also be that those with lower parent education levels may have less time for social media due to balancing school and potential work obligations to help support them. Age was the only identified protective factor against ESMU. Results from the current study reported that college students who were younger were at decreased odds of ESMU. Previously, age has been demonstrated to be associated with ESMU and addictive social media use, with younger adults using social media more than older adults (Andreassen et al., 2017; Bonsaksen et al., 2024; Mathis et al., 2022). Sociodemographic predictors of ESMU provide valuable insights to effectively target ESMU among college students.

College-specific characteristics including enrollment status, class format, current residence, and fraternity or sorority membership were also identified as predictors of ESMU. In the current cannabis use model only, college students that were enrolled part-time were at increased odds of ESMU compared to full-time students. This relationship has not been previously investigated, indicating a newly identified relationship between enrollment status and ESMU. In both the current cannabis use and cannabis use risk models, students enrolled in entirely online classes were at increased odds of reporting ESMU compared to entirely in-person classes. Additionally, those enrolled in hybrid classes were at increased odds of ESMU compared to those enrolled in entirely in-person classes. In support of these findings, previous research has found that college students are more likely to engage in multi-tasking behaviors, such as using social media sites during entirely online classes compared to entirely in-person classes (Lepp et al., 2019). Within the current study’s current cannabis use model, college students who lived off-campus or at a family member’s home were at increased risk for ESMU compared to students living in campus housing. In the cannabis use risk model, living in a family member’s home was found to be the only predictor of ESMU compared to those who lived in campus housing. Among college students, previous research has indicated that social media use may provide students living off-campus with opportunities to make friends and feel a sense of belonging (Yang et al., 2025). Based on these findings, students living off-campus or in a family member’s home may excessively use social media as a means to socially engage and connect with other students living on campus. In both the current cannabis use and cannabis use risk models, fraternity or sorority membership was also associated with ESMU with members having an increased odds of reporting ESMU compared to non-members. The use of social media platforms for fraternities or sororities to engage with members and provide internal news has been previously identified (Taylor & McArdle, 2018). Although speculative, fraternity or sorority members may engage in ESMU to stay current on internal chapter news or to socially engage with other members. To comprehensively understand the college related factors predicting ESMU among students, further research is needed. The current study expanded upon previous research presenting sociodemographic and college factors related to ESMU among college students.

Concerning cannabis use covariates, this study found that female college students had higher reports of current cannabis use than male college students. Previously, research demonstrated that male college students tended to report higher percentages of cannabis use compared to female college students (Keith et al., 2015; McCabe et al., 2007); however, more recently, cannabis use rates in women have increased to comparable rates of men (Martin et al., 2025; Patrick et al., 2024a). Current cannabis use was also associated with participants’ race and ethnicity with non-Hispanic Other/Multiracial and non-Hispanic White college students reporting higher percentages than any other race or ethnicity. Prior research indicates White college students have a higher frequency of use within the past 30 days (Keith et al., 2015); however, odds of cannabis use and dependence are higher in Black adults (Wu et al., 2016). As indicated from a longitudinal study, cannabis use among White adults typically peaks earlier than Black adults, which may provide reasoning for the current study’s results (Patrick et al., 2021). Participant age and undergraduate year were both found to be associated with current cannabis use. The current study indicated higher percentages of current cannabis use as undergraduate year increased, which is similar to other research that reported undergraduate students in their senior year had higher percentages of regular or frequent cannabis use than other undergraduate years (Keith et al., 2015). Regarding age findings, previous research has indicated that the peak age for cannabis use among adults is between 18–20 years old, which aligns with the results presented (Patrick et al., 2021). This study found parent’s highest education level to be associated with current cannabis use with higher percentages of current cannabis use among participants with a parent’s education of some college and undergraduate or graduate level degrees compared to parents with a ≤high school graduate or equivalent education level. These findings are in alignment with previous research among young adults indicating that a parent’s highest education level of some college had significantly higher odds of cannabis use than those with a lower parent education level (Patrick et al., 2016). College students enrolled in entirely in-person classes and living in off-campus housing reported higher rates of current cannabis use. There is limited research on class format and residence with cannabis use among college students. It can be speculated that students taking in-person classes and living off-campus are perceiving their peers using cannabis more frequently which may impact their cannabis use (Kenyon et al., 2024; Patrick et al., 2016). Within this study, being a member of a fraternity or sorority was associated with current cannabis use. Fraternity or sorority membership has previously been identified as a risk factor for cannabis use compared to non-members (Keith et al., 2015; Middleton et al., 2023). The findings from this study fill gaps in the characteristics of college students using cannabis.

This study has several strengths including the use of 2022–2023 ACHA-NCHA III survey data, which involves a recent, large, nationally representative sample of U.S. college students aged 18–24 years old. However, there are several associated limitations in this study. First, the ACHA-NCHA III is a cross-sectional survey, and causal or longitudinal associations cannot be inferred from these data. Subsequently, the identified associations could be bidirectional. Secondly, the ACHA-NCHA III only includes self-reported data by participants, which may have introduced recall bias or social desirability biases. Cannabis use, cannabis use risks, and time spent on social media may have been underreported by participants. The time spent using social media should be objectively measured in future research to eliminate recall biases. The study design of this secondary dataset may introduce potential bias in that the use of a larger dataset could derive significant, inconsequential effects such as trivial significant findings with small effect sizes (Kaplan et al., 2014). These findings should be interpreted with caution. Additionally, due to the use of a national dataset’s measures on cannabis use and social media use were limited. The time cut-points for the social media measure were pre-determined by the ACHA-NCHA. Due to this, the ESMU measure was assessed as <16 h/week, which differed from the <14 h/week presented in previous research (Primack et al., 2017; Sampasa-Kanyinga & Lewis, 2015). There were no measures on methods of cannabis use, the cultivar of the Cannabis plant used, and potency of cannabis used. Different cultivars of the Cannabis plant can produce subjective effects, with the Cannabis Sativa plant eliciting feelings of energy or alertness, and the Cannabis Indica plant generating feelings of tiredness or relaxation (Okey et al., 2023; Sholler et al., 2022). The cultivar of the cannabis used could potentially impact the motivation for ESMU. Availability of higher potency cannabis products has also increased due to cannabis legalization (ElSohly et al., 2016). The potency of the cannabis product used by college students could provide insight into a potential dose–response relationship with ESMU. Future research should explore cannabis behaviors and product use to further understand these relationships. Additional social media use measures such as the type of social media site used, and time spent on specific social media sites could provide vital information for preventive interventions to target ESMU among college students. Lastly, due to the specific population of U.S. college students ages 18–24 years, there are limitations to the generalizability of this study. Due to the voluntary nature of the ACHA-NCHA III for institutions and participants, selection bias can be introduced which potentially limits the generalizability of these results outside of participating institutions.

## 5. Conclusions

This study presented findings that demonstrated an association between current cannabis use and cannabis use risk with ESMU among U.S. college students. In addition to identifying a unique relationship, these results provided essential information to address cannabis use and ESMU among college students. With the increased odds of ESMU indicated for college students reporting current cannabis use and moderate or high cannabis use risk levels, social media messaging for cannabis use or cannabis use disorder could successfully reach this population. While pro-cannabis content on social media increases intentions to use cannabis among college students, anti-cannabis content can decrease intentions of use (Willoughby et al., 2024). Social media provides a convenient and accessible platform to educate college students on the risks and effects of continued cannabis use. Additionally, messaging about the odds of cannabis use with ESMU are necessary to increase knowledge regarding the dual use between cannabis and social media, and the potential effects. This study also provided predictors for ESMU among college students, which can help create targeted messaging and interventions for college students. The role of social media platforms in ESMU should not be forgotten. TikTok is one of the only social media platforms to allow users to set limits on daily usage and alerts users if they’ve been scrolling for too long (Mlot, 2022). The use of time limits and messaging on social media sites could be potential strategies to decrease ESMU among cannabis users. As the results presented in this study are preliminary, further longitudinal research is necessary to fully comprehend the relationship between cannabis use and ESMU among college students.

## Figures and Tables

**Table 1:** Sociodemographic Characteristics Overall and Based on Current Cannabis Use Among U.S. College Students 18–24 Years Old, 2022–2023 ACHA-NCHA III.

Characteristics		Past 30-Day Cannabis Use		
	Overall	No Current Use	Current Use	*p*-Value
	*n* (%) ^a^	*n* (%) ^a^	*n* (%) ^a^	
Age, *M* (SD)	19.9 (1.4)	19.8 (1.4)	20.0 (1.4)	<0.001
Sex				<0.001
Male	18,510 (28.5)	14,458 (78.1)	4052 (21.9)	
Female	46,542 (71.5)	35,510 (76.3)	11,032 (23.7)	
Race/Ethnicity				<0.001
Non-Hispanic White	37,000 (56.9)	27,671 (74.8)	9329 (25.2)	
Non-Hispanic Black	2892 (4.4)	2311 (79.9)	581 (20.1)	
Non-Hispanic Asian	8787 (13.5)	7568 (86.1)	1219 (13.9)	
Non-Hispanic Other/Multiracial	9958 (15.3)	7356 (73.9)	2602 (26.1)	
Hispanic	6415 (9.9)	5062 (78.9)	1353 (21.1)	
Undergraduate Year				<0.001
1st year	20,632 (31.7)	16,663 (80.8)	3969 (19.2)	
2nd year	16,138 (24.8)	12,366 (76.6)	3772 (23.4)	
3rd year	15,787 (24.3)	11,862 (75.1)	3925 (24.9)	
≥4th year	12,495 (19.2)	9077 (72.6)	3418 (27.4)	
Enrollment Status				0.066
Full-time	62,626 (96.3)	48,142 (76.9)	14,484 (23.1)	
Part-time	2426 (3.7)	1826 (75.3)	600 (24.7)	
Class Format				<0.001
Entirely in-person	42,953 (66.0)	32,597 (75.9)	10,356 (24.1)	
Entirely online	1667 (2.6)	1326 (79.5)	341 (20.5)	
Hybrid (in-person and online)	20,432 (31.4)	16,045 (78.5)	4387 (21.5)	
Fraternity/Sorority Membership				<0.001
No	60,025 (92.3)	46,627 (77.7)	13,398 (22.3)	
Yes	5027 (7.7)	3341 (66.5)	1686 (33.5)	
Current Residence				<0.001
Campus housing	33,843 (52.0)	26,307 (77.7)	7536 (22.3)	
Off-campus housing	20,309 (31.2)	14,218 (70.0)	6091 (30.0)	
Family member home	10,900 (16.8)	9443 (86.6)	1457 (13.4)	
Parent’s Education Level				<0.001
≤High school graduate or equivalent	12,845 (19.7)	10,273 (80.0)	2572 (20.0)	
Some college	9869 (15.2)	7620 (77.2)	2249 (22.8)	
Undergraduate degree	18,666 (28.7)	14,278 (76.5)	4388 (23.5)	
Graduate degree	23,672 (36.4)	17,797 (75.2)	5875 (24.8)	

^a^ *n* refers to count and % refers to row percent, unless noted otherwise.

**Table 2:** Unadjusted and Adjusted Logistic Regression Model Results of the Association Between Current Cannabis Use and ESMU Among U.S. College Students 18–24 Years Old, 2022–2023 ACHA-NCHA III.

	ESMU (≥16 h) (*n* = 65,052)	Unadjusted Logistic Regression			Adjusted Logistic Regression		
	*n* (%) ^a^	OR	95% CI	*p*-Value	AOR	95% CI	*p*-Value
Past 30-Day Cannabis Use							
No Current Use	6315 (12.6)	Ref	Ref	Ref	Ref	Ref	Ref
Current Use	2260 (15.0)	1.22	1.16–1.28	<0.001	1.27	1.20–1.34	<0.001
Age, *M* (SD)	19.8 (1.4)	-	-	-	0.97	0.94–0.99	0.011
Sex							
Male	2298 (12.4)	-	-	-	Ref	Ref	Ref
Female	6277 (13.5)	-	-	-	1.06	1.00–1.11	0.034
Race/Ethnicity							
Non-Hispanic White	4631 (12.5)	-	-	-	Ref	Ref	Ref
Non-Hispanic Black	518 (17.9)	-	-	-	1.49	1.35–1.65	<0.001
Non-Hispanic Asian	1186 (13.5)	-	-	-	1.15	1.07–1.23	<0.001
Non-Hispanic Other/Multiracial	1351 (13.6)	-	-	-	1.09	1.02–1.17	0.008
Hispanic	889 (13.9)	-	-	-	1.01	0.93–1.10	0.751
Undergraduate Year							
1st year	2789 (13.5)	-	-	-	Ref	Ref	Ref
2nd year	2148 (13.3)	-	-	-	0.97	0.90–1.03	0.322
3rd year	2005 (12.7)	-	-	-	0.93	0.85–1.01	0.080
≥4th year	1633 (13.1)	-	-	-	0.98	0.88–1.08	0.645
Enrollment Status							
Full-time	8176 (13.1)	-	-	-	Ref	Ref	Ref
Part-time	399 (16.4)	-	-	-	1.17	1.05–1.32	0.007
Class Format							
Entirely in-person	5048 (11.8)	-	-	-	Ref	Ref	Ref
Entirely online	318 (19.1)	-	-	-	1.60	1.40–1.83	<0.001
Hybrid (in-person and online)	3209 (15.7)	-	-	-	1.35	1.29–1.42	<0.001
Fraternity/Sorority Membership							
No	7840 (13.1)	-	-	-	Ref	Ref	Ref
Yes	735 (14.6)	-	-	-	1.20	1.10–1.30	<0.001
Current Residence							
Campus housing	4186 (12.4)	-	-	-	Ref	Ref	Ref
Off-campus housing	2682 (13.2)	-	-	-	1.06	1.00–1.13	0.046
Family member home	1707 (15.7)	-	-	-	1.18	1.11–1.27	<0.001
Parent’s Education Level							
≤High school graduate or equivalent	1881 (14.6)	-	-	-	Ref	Ref	Ref
Some college	1451 (14.7)	-	-	-	1.23	1.15–1.32	<0.001
Undergraduate degree	2532 (13.6)	-	-	-	1.23	1.15–1.32	<0.001
Graduate degree	2711 (11.5)	-	-	-	1.18	1.11–1.25	<0.001

Abbreviations: OR, odds ratio; CI, confidence interval; AOR, adjusted odds ratio; M, mean; SD, standard deviation; Ref, reference. ^a^ *n* refers to count and % refers to column percent, unless noted otherwise.

**Table 3:** Unadjusted and Adjusted Logistic Regression Model Results of the Association Between Cannabis Use Risk and ESMU Among U.S. College Students 18–24 Years Old, 2022–2023 ACHA-NCHA III.

	ESMU (≥16 h) (*n* = 25,431)	Unadjusted Logistic Regression			Adjusted Logistic Regression		
	*n* (%) ^a^	OR	95% CI	*p*-Value	AOR	95% CI	*p*-Value
SSIS Cannabis Score							
Low Cannabis Use Risk (0–3)	1574 (13.2)	Ref	Ref	Ref	Ref	Ref	Ref
Moderate Cannabis Use Risk (4–26)	1957 (15.5)	1.21	1.12–1.30	<0.001	1.21	1.12–1.30	<0.001
High Cannabis Use Risk (27–39)	186 (21.3)	1.78	1.50–2.11	<0.001	1.82	1.53–2.16	<0.001
Age		-	-	-	0.94	0.90–0.98	0.002
Sex							
Male	914 (13.6)	-	-	-	Ref	Ref	Ref
Female	2803 (15.0)	-	-	-	1.08	0.99–1.17	0.059
Race/Ethnicity							
Non-Hispanic White	2217 (14.0)	-	-	-	Ref	Ref	Ref
Non-Hispanic Black	190 (20.4)	-	-	-	1.52	1.28–1.80	<0.001
Non-Hispanic Asian	333 (15.5)	-	-	-	1.17	1.03–1.32	0.017
Non-Hispanic Other/Multiracial	639 (15.1)	-	-	-	1.08	0.98–1.19	0.119
Hispanic	338 (14.5)	-	-	-	0.95	0.83–1.08	0.411
Undergraduate Year							
1st year	1020 (16.1)	-	-	-	Ref	Ref	Ref
2nd year	938 (15.0)	-	-	-	0.96	0.86–1.07	0.413
3rd year	922 (13.5)	-	-	-	0.90	0.79–1.03	0.109
≥4th year	837 (13.9)	-	-	-	0.98	0.83–1.15	0.768
Enrollment Status							
Full-time	3547 (14.5)	-	-	-	Ref	Ref	Ref
Part-time	170 (17.4)	-	-	-	1.14	0.96–1.36	0.146
Class Format							
Entirely in-person	2230 (13.1)	-	-	-	Ref	Ref	Ref
Entirely online	129 (21.4)	-	-	-	1.74	1.40–2.15	<0.001
Hybrid (in-person and online)	1358 (17.5)	-	-	-	1.38	1.28–1.49	<0.001
Fraternity/Sorority Membership							
No	3299 (14.5)	-	-	-	Ref	Ref	Ref
Yes	418 (15.7)	-	-	-	1.18	1.06–1.33	0.003
Current Residence							
Campus housing	1768 (14.3)	-	-	-	Ref	Ref	Ref
Off-campus housing	1405 (13.9)	-	-	-	1.01	0.92–1.10	0.915
Family member home	544 (18.3)	-	-	-	1.24	1.10–1.39	<0.001
Parent’s Education Level							
≤High school graduate or equivalent	707 (16.3)	-	-	-	Ref	Ref	Ref
Some college	619 (15.9)	-	-	-	1.20	1.07–1.33	0.001
Undergraduate degree	1109 (14.9)	-	-	-	1.17	1.05–1.30	0.004
Graduate degree	1282 (13.1)	-	-	-	1.13	1.04–1.24	0.006

Abbreviations: OR, odds ratio; CI, confidence interval; AOR, adjusted odds ratio; SSIS, Substance Specific Involvement Score; Ref, reference. ^a^ *n* refers to count and % refers to column percent, unless noted otherwise.

## Data Availability

The data that support the findings of this study are available for request from the American College Health Association’s (ACHA’s) National College Health Assessment (NCHA) website. Restrictions apply to the availability of these data, which were used under license for this study. Data are from the ACHA-NCHA with the permission of the ACHA-NCHA.

## Abbreviations

The following abbreviations are used in this manuscript:

ESMU	Excessive social media use
ACHA	American College Health Association
NCHA	National College Health Assessment
OR	Odds ratio
AOR	Adjusted odds ratio
